# Ticagrelor and gut microbiota: Absence of pharmacokinetic interaction *in vitro* and *in vivo* studies

**DOI:** 10.1371/journal.pone.0359660

**Published:** 2026-10-01

**Authors:** Pan Qu, Yongchao Gao, Xiaojie Li, Li Yang, Jie Tang, Manyun Chen, Yicheng Wang, Yilei Peng, Junyi Jiang, Weihua Huang, Wei Zhang, Yali Liu, Yali Ou

**Affiliations:** 1 Engineering Research Center of Applied Technology of Pharmacogenomics (Ministry of Education, China), Hunan Key Laboratory of Pharmacomicrobiomics, Department of Clinical Pharmacology, Xiangya Hospital, Central South University, Changsha, China; 2 Department of Pharmacy, Hunan Prevention and Treatment Institute for Occupational Diseases, Affiliated Prevention and Treatment Institute for Occupational Diseases of University of South China, Changsha, China; 3 The First Affiliated Hospital of Shantou University Medical College, Shantou, China; 4 Department of Pharmacy, Hunan Provincial People's Hospital, the First Affiliated Hospital of Hunan Normal University, Changsha, China; 5 Department of Cardiovascular, Xiangya Hospital, Central South University, Changsha, China; Mie University Hospital: Mie Daigaku Igakubu Fuzoku Byoin, JAPAN

## Abstract

Ticagrelor, a commonly used antiplatelet medication, has been found to exhibit considerable inter-individual pharmacodynamic variability. Recent studies have revealed a close relationship between pharmacokinetics and pharmacodynamics, both of which can be influenced by gut microbiota. In this study, we explored whether the pharmacokinetics of ticagrelor is mediated by intestinal flora. We enrolled 22 healthy participants to assess the clinical pharmacokinetic profiles of ticagrelor and its metabolite AR-C124910XX after a single oral dose of ticagrelor. The fecal microbial compositions of individuals who exhibited distinct pharmacokinetic parameters were analyzed using 16S rRNA gene sequencing. Additionally, we conducted experiments involving the co-incubation of ticagrelor with fecal bacteria and a systematic exploration of the pharmacokinetics of ticagrelor in pseudo-germ-free (PGF) rats to illustrate the role of gut microbiota in ticagrelor metabolism, both *in vitro* and *in vivo*. Our findings revealed a three-fold difference in the bioavailability of ticagrelor among individuals. However, no difference was found in the composition of gut microbiota among individuals with different pharmacokinetic parameters. Quantitative analysis showed that gut microbiota cannot directly metabolize ticagrelor. Furthermore, there were no significant differences in AUC_(0–48)_, AUC_(0-∞),_ and C_max_ between ticagrelor and AR-C124910XX in PGF rats compared to normal control, either by single intragastric or intravenous administration. In conclusion, these findings suggest that gut microbiota has no impact on the pharmacokinetics of ticagrelor.

## 1. Introduction

Ticagrelor, a cyclopentyltriazole compound, is a selective adenosine diphosphate receptor antagonist with a dual pathway inhibitory effect on platelets [1,2]. The latest American College of Cardiology and American Heart Association guidelines recommend ticagrelor as a first-line agent for the treatment of acute ST-segment elevation myocardial infarction (STEMI) [3]. However, there are significant individual differences in the clinical efficacy of ticagrelor. The PLATO subgroup study found that approximately 3%−27% of patients had a low response to ticagrelor (platelet reactivity index > 50%) [4]. Gene polymorphisms have limited effects on ticagrelor: P2RY12, P2RY1, and ITGB3 polymorphisms do not affect the inhibition of platelet aggregation by ticagrelor [5], and CYP2C19 and ABCB1 polymorphisms do not affect the clinical efficacy of ticagrelor [6]. Therefore, the factors that contribute to the individual differences in ticagrelor efficacy need to be further explored.

Studies have shown that changes in pharmacokinetics can alter drug efficacy and side effects [7]. Gut microbiota has been identified as an important factor in the regulation of drug pharmacokinetics and pharmacodynamics, as well as individual variability in drug response [8]. Recent studies have demonstrated that gut microbiota may affect drug biotransformation including absorption, distribution, metabolism, and excretion, which in turn affects pharmacodynamics, such as the ability of intestinal bacteria to convert drugs into biologically active substances (Sulfasalazine, lovastatin, and ginsenoside Rb1), inactive substances (Ranitidine and metronidazole), and toxic metabolites, *etc* [8–12]. Currently, the mechanisms of drug pharmacokinetics influenced by gut microbiota can be divided into three main categories: (1) Direct metabolism [13]; (2) Bioaccumulation [14]; (3) Gut microbiota metabolites alter drug pharmacokinetics by affecting metabolic enzymes, transporters [15]. Previous research has tentatively established a potential relationship between the plasma concentration of ticagrelor and its metabolite AR-C124910XX and gut microbiota [16]. However, the effects of gut microbiota on the pharmacokinetics of ticagrelor are not well explored.

Herein, with genetic polymorphism almost ruled out, we intended to explore whether the gut microbiota affects the pharmacokinetics of ticagrelor. By enrolling healthy subjects, we found significant individual differences in the pharmacokinetic profiles of ticagrelor and its metabolite AR-C124910XX, but no differences in the composition of gut microbiota among individuals with distinct pharmacokinetic parameters. Ticagrelor couldn’t be metabolized by gut microbiota *in vitro*. By comparing the plasma concentration of ticagrelor and AR-C124910XX in pseudo-germ-free (PGF) rats with control rats, we confirmed that gut microbiota had no influence on the pharmacokinetics of ticagrelor *in vivo*. Our results implied that gut microbiota may not be responsible for the pharmacokinetic differences in ticagrelor.

## 2. Materials and methods

### 2.1. Materials and reagents

Ticagrelor tablets (AstraZeneca Co., Ltd.), ticagrelor (98% purity, Aladdin Co., Ltd.), and AR-C124910XX (Push Bio-technology Co., Ltd.). Carbamazepine (98% purity) was provided by the National Institutes for Food and Drug Control (Beijing, China) and was selected as the internal standard (IS) for the pharmacokinetics experiment. Ampicillin, metronidazole, vancomycin hydrochloride, and neomycin sulphate were purchased from Sangon Biotech Co., Ltd (Shanghai, China). Gifu Anaerobic Medium, modified (mGAM) was purchased from Nissui Co., Ltd (Tokyo, Japan). QIAamp^®^ PowerFecal^®^ Pro DNA Kit was purchased from QIAGEN (Hilden, Germany).

Methanol was purchased from Merck & Co., Inc. (Kenilworth, NJ, USA). Formic acid was purchased from ANPEL Laboratory Technologies Inc.(Shanghai, China). Unless otherwise stated, all reagents were of analytical grade.

### 2.2. Human studies

The recruitment period for this study was conducted from June 1, 2020, to August 31, 2020. All participants in this study have signed the informed consent form, and the study protocol has been approved by the Institutional Review Board of the First Affiliated Hospital of Shantou University Medical College (ethical number: 2019005). All subjects were 19–36 years old and in good health. The health status of subjects was judged based on medical history, physical examination, and routine laboratory tests. These subjects had not taken any medications (prescription or nonprescription) for 2 weeks before the study and did not anticipate any need for medication during the study. The clinical details of the included cases are listed in S1 Table.

Twenty-two subjects received oral 90 mg ticagrelor with 240 mL water, which was to be swallowed whole, not swallowed or chewed. Plasma samples were collected at designated times for pharmacokinetic analysis. For the subjects, blood samples were collected before drug administration (0 h), and at 20, 40 min, 1, 1.5, 2, 2.5, 3, 4, 6, 8, 12, 24, 36, and 48 h after dosing. 4 mL of blood was collected into a labeled K2-EDTA anticoagulation tube, which was centrifuged at 2,000 × *g* at 4 °C for 10 min. The plasma supernatants were stored below −80 °C until assay. Hierarchical clustering analysis was applied to identify subject clusters with different pharmacokinetic parameters.

### 2.3. 16S rRNA sequencing for human gut microbiota

The compositions of the intestinal flora of fecal samples from 22 volunteers were examined by 16S rRNA sequencing analysis. The total DNA was isolated from stool samples. After extracting the total DNA of the samples, PCR amplification was performed and the products were purified, quantified, and homogenized to form sequencing libraries. The constructed libraries were first subjected to quality control, and the libraries that passed the quality control were sequenced by Illumina NovaSeq. The results are stored in FASTQ (fq) file format, which contains the sequence information and sequencing quality information. High-quality clean tags were obtained by splicing and filtering the original Reads, followed by subsequent analysis using the EasyAmplicon [17] pipeline. Then the data were subjected to basic analysis and the processed tags were denoised with “unoise3” to obtain single-base precision ASVs, which were dechimerized, deplasmidized, and non-bacterialized. Species annotation, diversity analysis, and species composition analysis of ASV representative sequences were performed using the Silva database. The *α*-diversity was computed by the R package ‘vegan’, and the *β*-diversity was calculated by principal coordinate analysis (PCoA) at the ASV level. The linear discriminant analysis (LDA) effect size (LEfSe) method was applied to explore biomarker features.

### 2.4. Gut microbiota preparation

An appropriate amount of human or rat fresh fecal sample was suspended well in cold physiological saline (w/v, 1:20) before vortexing for adequate mixing and then centrifuged at 500 rpm for 5 min at 4 °C. The resultant fecal supernatant was decanted and centrifuged at 4,000 rpm for 30 min at 4 °C, then the precipitate was re-suspended with 2.5 mL of mGAM to produce gut microbiota suspension. Stocks were generated by adding glycerol to the gut microbiota suspension (final concentration of glycerol, 20%) and were anaerobically stored in −80 °C freezer [18].

### 2.5. Ticagrelor preparation and co-incubation with fecal samples

Gut microbiota stock was activated with mGAM broth (1:20). After incubation for 24 h, the medium was centrifuged at 4,000 rpm for 30 min at 4 °C. The supernatant was discarded and the precipitate was re-suspended in mGAM broth as the gut microbiota work solution. The biotransformation of the ticagrelor by the gut microbiota was performed in an incubation system containing 500 μL of the gut microbiota work solution and 5 μL of ticagrelor solution (1.07 μg/mL, solution in dimethyl sulphoxide). Negative control was performed in an incubation system containing 500 μL of the mGAM broth and 5 μL of ticagrelor solution. The incubation system was anaerobically incubated at 37 °C for 24 h and stopped with the addition of 600 μL cold methanol including IS (carbamazepine) in a 200 μL incubation system. The samples were centrifuged at 14,000 rpm for 10 min and detected by UPLC-MS/MS.

### 2.6. Animals

SD rats (specific pathogen-free (SPF), Certificate No. SCXK2019–0004), 250 ± 20 *g*, were purchased from Hunan SJA Laboratory Animal Co., Ltd (Changsha, China). The animals were maintained in the SPF animal facilities of the Animal Experimental Center of Central South University and natural light-dark cycle conditions with controlled temperature (22 ± 2°C), food and drinking water supplied unrestricted. All animal work was done in accordance with the National Institutes of Health’s Guide for the Use and Care of Laboratory Animals and was approved by the Institutional Review Board of Central South University (Hunan, China. permission numbers: CSU-2022-0001-0017).

Rats were acclimated to the laboratory for 1 week before the experiment and randomized into two treatment groups: control group and PGF group (pseudo-germ-free) (n = 8). Rats in the PGF group were gavage with an antibiotic cocktail (100 mg/kg ampicillin, 100 mg/kg neomycin sulfate, 100 mg/kg metronidazole, and 50 mg/kg vancomycin), and provided with an antibiotic cocktail (0.25 g/L ampicillin, 0.25 g/L neomycin sulfate, 0.25 g/L metronidazole and 0.125 g/L vancomycin) in drinking water for 2 weeks to deplete the intestinal flora. To validate the pseudo-sterile rat model, rat feces were collected and fecal DNA was extracted using a QIAamp^®^ PowerFecal^®^ Pro DNA kit. The amount of intestinal microbiota DNA was measured using a BioSpec-nano spectrophotometer to assess the stability and validity of the pseudo-sterility model [19].

All surgeries were performed under anesthesia (isoflurane). Animals were euthanized when humane endpoints were reached, which included abnormal posture, failure to groom, lethargy, impaired ambulation or paralysis, weight loss exceeding 20% normal body weight, and the inability to voluntarily access food and water. Rats reaching these endpoints were euthanized immediately via CO2 inhalation followed by cervical dislocation to minimize suffering and distress. Animals were monitored daily for health and behaviour, and no animals died before meeting euthanasia criteria. All research staff received training in animal anaesthesia, animal surgery, experimental procedures, and monitoring/nursing. All animal welfare considerations were taken to minimize suffering and distress, including frequent monitoring and immediate euthanasia at endpoint.

### 2.7. Pharmacokinetic experiments

Rats were fasted 12 hours before the pharmacokinetic experiments. 18 mg/kg of ticagrelor (0.5% CMC-Na solution as solvent) was administered to the rats by intragastric gavage. Blood samples were collected from the tail vein at 0, 0.25, 0.5, 1, 2, 3, 4, 6, 7.5 and 24 h. Additionally, 3 mg/kg of ticagrelor (2% Tween 80 solution as solvent) was intravenously administered to the rats. The blood samples were collected from the tail vein at 0, 0.25, 0.5, 1, 2, 3, 4, 5, 6, and 24 h. The blood sample was centrifuged at 4,000 rpm at 4 °C for 15 min and supernatant plasma was collected and stored at −80 °C for further analysis.

### 2.8. Quantification of plasma ticagrelor and AR-C124910XX

Compounds were extracted from plasma by albumen precipitation method. The concentration of the ticagrelor and AR-C124910XX was determined on the AB Sciex Qtrap 6500 UPLC-MS/MS system (AB Sciex, Massachusetts, USA). Targeted analytes were separated using an ACE Excel 5 Super C18 column (150 mm x 2.1 mm, Radnor, PA, USA); the flow rate was maintained at 0.4 mL/min. The mobile phase consisted of solvent A (deionized water with 0.1% formic acid) and solvent B (methanol). A gradient program was used for the HPLC separation: 0–0.5 min (30% A); 0.5–1.5 min (30%−5% A); 1.5–2.0 min (5% A); 2.0–2.5 min (5%−30% A); 2.5–4.0 min (30% A). The oven temperature was maintained at 40℃ and the injection volume was set at 2 μL. The mass spectrometer was operated in positive ion mode through multiple-reaction monitoring. The precursor-product ion pairs monitored were m/z 523.4 → 152.9 for ticagrelor (DP 185 V, CE 46 eV), m/z 479.3 → 153.2 for AR-C124910XX (DP 155 V, CE 44 eV), and m/z 237.1 → 194.1 for carbamazepine (DP 130 V, CE 15 eV). Data were collected and processed using AB Sciex Analyst 1.4.2 data collection and integration software.

### 2.9. Statistical analysis

Pharmacokinetic parameters were analyzed by DAS 2.0 software (Chinese Pharmacological Association, China) using non-compartmental analysis. All data were represented as mean ± standard deviation (SD). The significance between the two groups was performed by using the unpaired two-tailed t-test or Mann-Whitney test, and *p* < 0.05 indicated that the difference was statistically significant.

## 3. Results

### 3.1. Individual variability in clinical pharmacokinetics of ticagrelor

To evaluate the population pharmacokinetic profile of ticagrelor in Chinese healthy individuals, 22 healthy individuals were recruited and received a single oral dose of ticagrelor (90 mg), then measured by UPLC-MS/MS. The plasma concentration-time curves of ticagrelor and AR-C124910XX within 48 h in healthy subjects are shown in Fig 1, and the mean pharmacokinetic parameters are summarized in Table 1. For ticagrelor, there was a 3-fold difference between individuals in AUC_(0–48)_ (1,429 ng·h/mL −5,582 ng·h/mL) and AUC_(0-∞)_ (1,568 ng·h/mL −5,746 ng·h/mL). At the same time, we found a 7-fold difference in the C_max_ of ticagrelor (137 ng/mL −953 ng/mL). The AUC_(0–48)_, AUC_(0-∞)_, and C_max_ of AR-C124910XX also showed significant individual differences. Altogether, these results indicated that large individual differences in the population pharmacokinetics of ticagrelor.

**Table 1:** Pharmacokinetic parameters of ticagrelor and AR-C124910XX in healthy subjects.

Parameters	Ticagrelor	AR-C124910XX
AUC_(0–48)_ (ng/mL*h)	3516 ± 1148	1790 ± 374.7
AUC_(0-∞)_ (ng/mL*h)	3592 ± 1159	1847 ± 395.6
C_max_ (ng/mL)	521.3 ± 171.4	174.9 ± 48.40
T_max_ (h)	2.341 ± 1.228	2.841 ± 1.084
t_1/2_ (h)	7.693 ± 1.330	9.355 ± 1.440
MRT_(0–48)_ (h)	8.425 ± 1.370	11.99 ± 1.409
MRT_(0-∞)_ (h)	9.343 ± 1.529	13.48 ± 2.092
CL (mL/h)	28.01 × 10^3^ ± 10.21 × 10^3^	51.33 × 10^3^ ± 13.43 × 10^3^
Vd (mL)	298.9 × 10^3^ ± 83.19 × 10^3^	682.0 × 10^3^ ± 158.1 × 10^3^

C_max_: maximum plasma concentration; T_max_: time to reach C_max_; AUC_(0–48)_ or AUC_(0-∞)_: area under the plasma concentration-time curve from zero to 48 h or infinity; t_1/2_: half-life of drug elimination; MRT: mean residence time; CL: clearance rate; Vd: apparent volume of distribution. Each value represents the mean ± SD.

**Fig 1:**
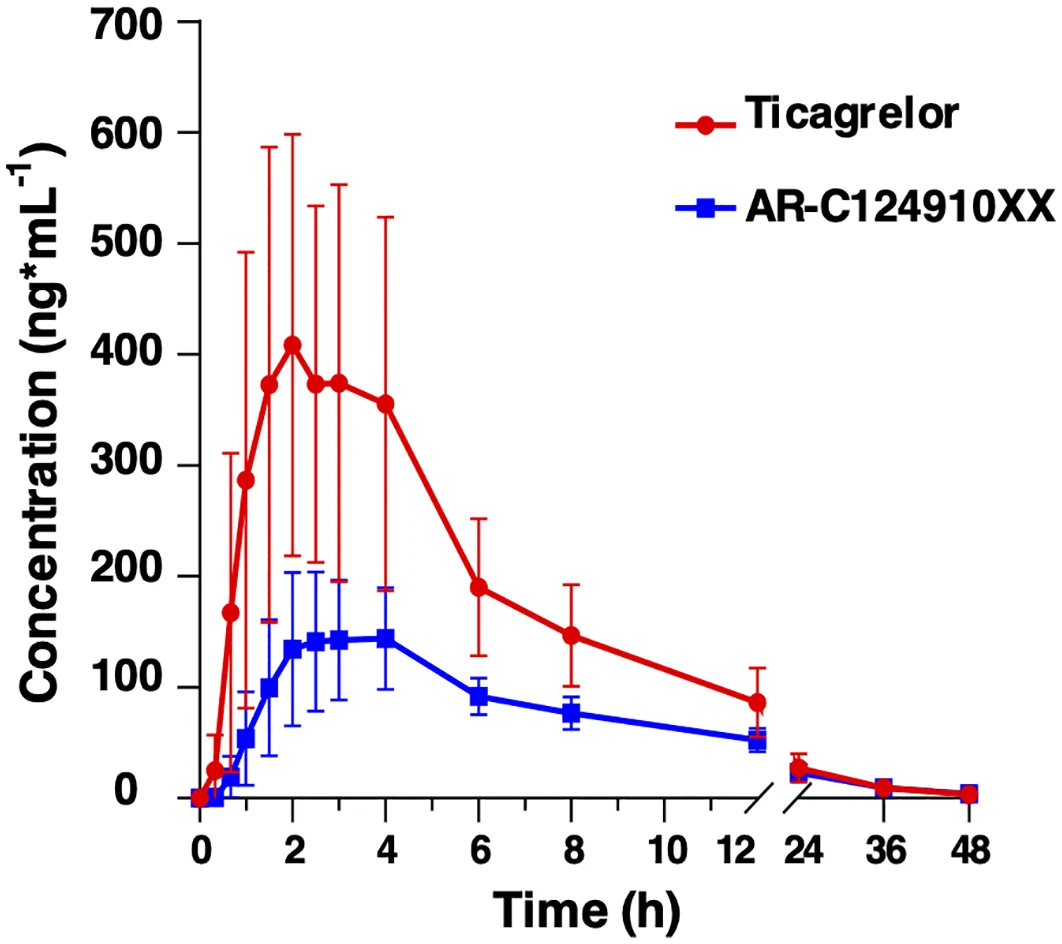
The plasma concentration-time curve of ticagrelor and AR-C124910XX within 48 h in healthy subjects. Data are represented as mean ± SD. n = 22.

### 3.2. Characteristics of gut microbiome of individuals with different pharmacokinetic parameters of ticagrelor

To explore whether individual differences in pharmacokinetic parameters of ticagrelor were related to gut microbiota. Subjects were divided into two groups based on ticagrelor pharmacokinetic parameters by using hierarchical clustering, Group H (with higher AUC, n = 12) versus Group L (with lower AUC, n = 10) (S1 Fig). Compared to Group L, the AUC_(0–48)_, AUC_(0-∞)_, C_max_, and t_1/2_ of ticagrelor were significantly higher than in Group H, with AUC_(0–48)_ increased by 79.51%, AUC_(0-∞)_ increased by 80.77%, C_max_ increased by 62.43% and t_1/2_ increased by 30.34%. However, CL was significantly reduced by 45.51%. In addition, the AUC_(0–48)_, AUC_(0-∞),_ and C_max_ of AR-C124910XX in Group H showed an increasing trend, but there was no statistical significance compared to Group L (S2 Fig, Table 2). These results suggested a significant increase in the bioavailability of ticagrelor for individuals in Group H.

**Table 2:** Pharmacokinetic parameters of ticagrelor and AR-C124910XX between the Group H and Group L.

Parameters	Ticagrelor		AR-C124910XX	
	H	L	H	L
AUC_(0–48)_ (ng/mL*h)	4434 ± 617.2	2470 ± 412.8****	1924 ± 290.9	1629 ± 414.2
AUC_(0-∞)_ (ng/mL*h)	4494 ± 642.7	2486 ± 418.1****	1991 ± 307.8	1676 ± 434.9
C_max_ (ng/mL)	631.7 ± 122.2	388.9 ± 120.6***	192.3 ± 48.60	153.9 ± 41.04
T_max_ (h)	1.958 ± 0.8649	2.800 ± 1.476	2.542 ± 0.7821	3.200 ± 1.316
t_1/2_ (h)	8.063 ± 0.8209	6.186 ± 2.335**	9.666 ± 1.562	8.981 ± 1.253
MRT_(0–48)_ (h)	8.988 ± 1.250	8.266 ± 1.193	11.97 ± 1.481	12.01 ± 1.395
MRT_(0-∞)_ (h)	9.636 ± 1.462	8.585 ± 1.369	13.62 ± 2.239	13.32 ± 2.008
CL (mL/h)	20.39 × 10^3^ ± 2834	37.42 × 10^3^ ± 8238****	46.30 × 10^3^ ± 7848	57.36 × 10^3^ ± 16.48 × 10^3^
Vd (mL)	236.2 × 10^3^ ± 34.12 × 10^3^	321.5 × 10^3^ ± 110.3 × 10^3^*	639.7 × 10^3^ ± 113.9 × 10^3^	732.7 × 10^3^ ± 193.0 × 10^3^

C_max_: maximum plasma concentration; T_max_: time to reach C_max_; AUC_(0–48)_ or AUC_(0-∞)_: area under the plasma concentration-time curve from zero to 48 h or infinity; t_1/2_: half-life of drug elimination; MRT: mean residence time; CL: clearance rate; Vd: apparent volume of distribution. Each value represents the mean ± SD. * *P* < 0.05*,* ** *P* < 0.01, *** *P* < 0.001, **** *P* < 0.0001 *vs.* the Group H.

Microbial DNA was isolated from fecal samples collected before ticagrelor administration and ed by 16S rRNA amplicon sequencing was performed to investigate the differences in gut microbiota between Group H and Group L. The results revealed no notable differences in *α*- (ACE, Chao, Simpson, and Shannon index) and *β*-diversities between Group H and Group L (Figs 2A and 2B). Additionally, we compared the abundance of bacteria taxa between Group H and Group L at phylum and genus levels. Compared with Group L, Firmicutes were significantly decreased in Group H on phylum level, while Proteobacteria were enriched (Figs 2C, E and F). At the genus level, only *Faecalibacterium* was found significantly enriched in Group L (*P* < 0.05, LDA scores > 4) (Figs 2D, E and F).

**Fig 2:**
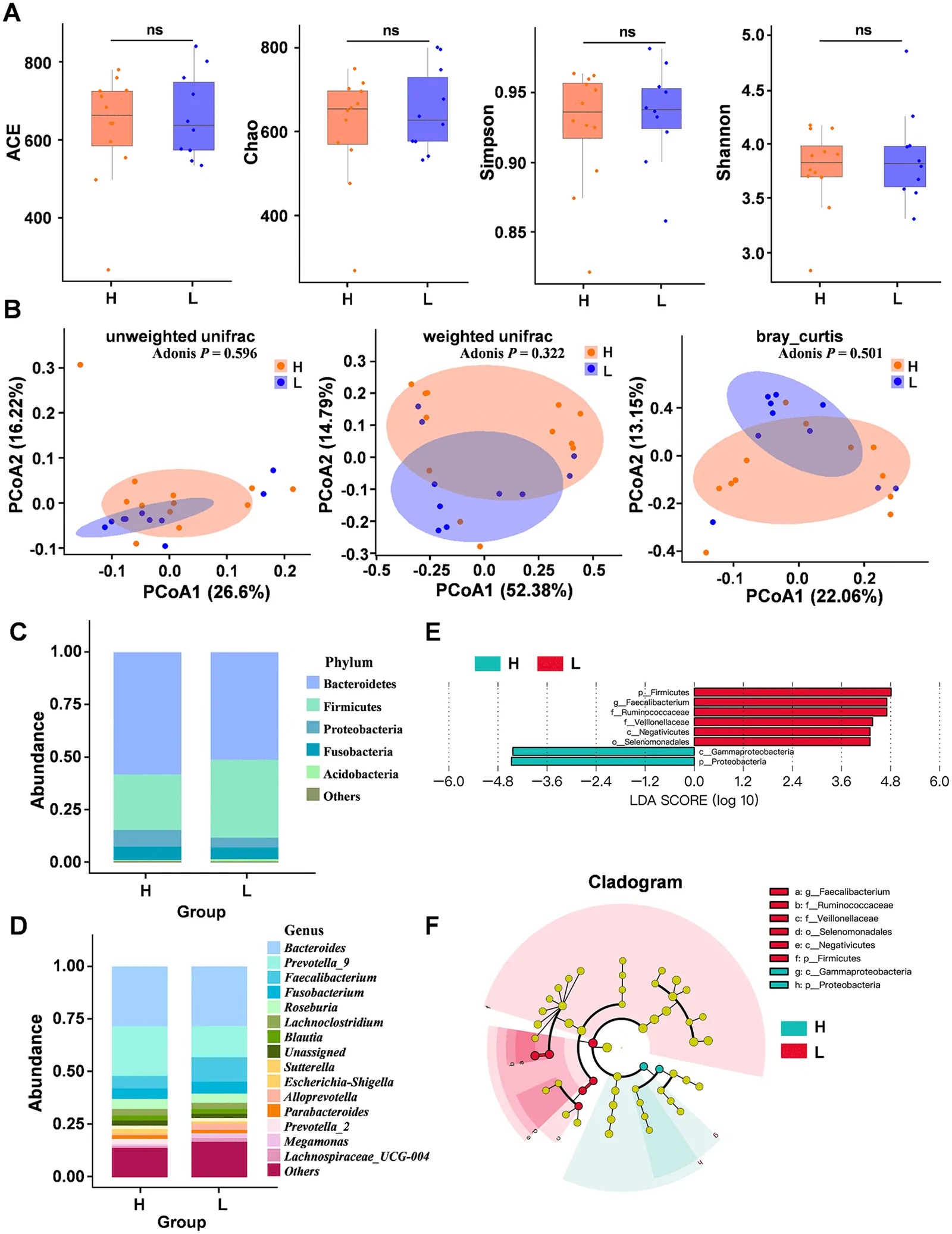
Comparison of gut microbiota between the Group H and Group L. (A) The alpha diversity boxplot (ACE, Chao, Simpson, and Shannon) in the Group H and Group L. Principal coordinates analysis (PCoA) (B) using unweighted unifrac distance, weighted unifrac distance, and bray-curtis metric distance of the Group H and Group L. Composition of the gut microbiome at phylum (C) and genus (D) taxonomic levels. (E) LEsSe analysis for gut microbiota between Group H and Group L (P < 0.05, LDA scores > 4). (F) Cladogram based on LDA scores. n = 12 for the Group H, n = 10 for the Group L. ns, no significance.

### 3.3. Absence of biotransformation of ticagrelor by gut microbiota *in vitro*

To investigate the bioconversion of ticagrelor mediated by gut microbiota, fecal bacteria from humans or rats were co-incubated with ticagrelor *in vitro* for 24 h, and ticagrelor was detected in the co-incubation system before (0 h) and after incubation (24 h). Compared to 0 h, there was no significant change in the amount of ticagrelor after 24 h of co-incubation with either human or rat gut microbiota (Fig 3). It indicated the absence of biotransformation of ticagrelor by gut microbiota.

**Fig 3:**
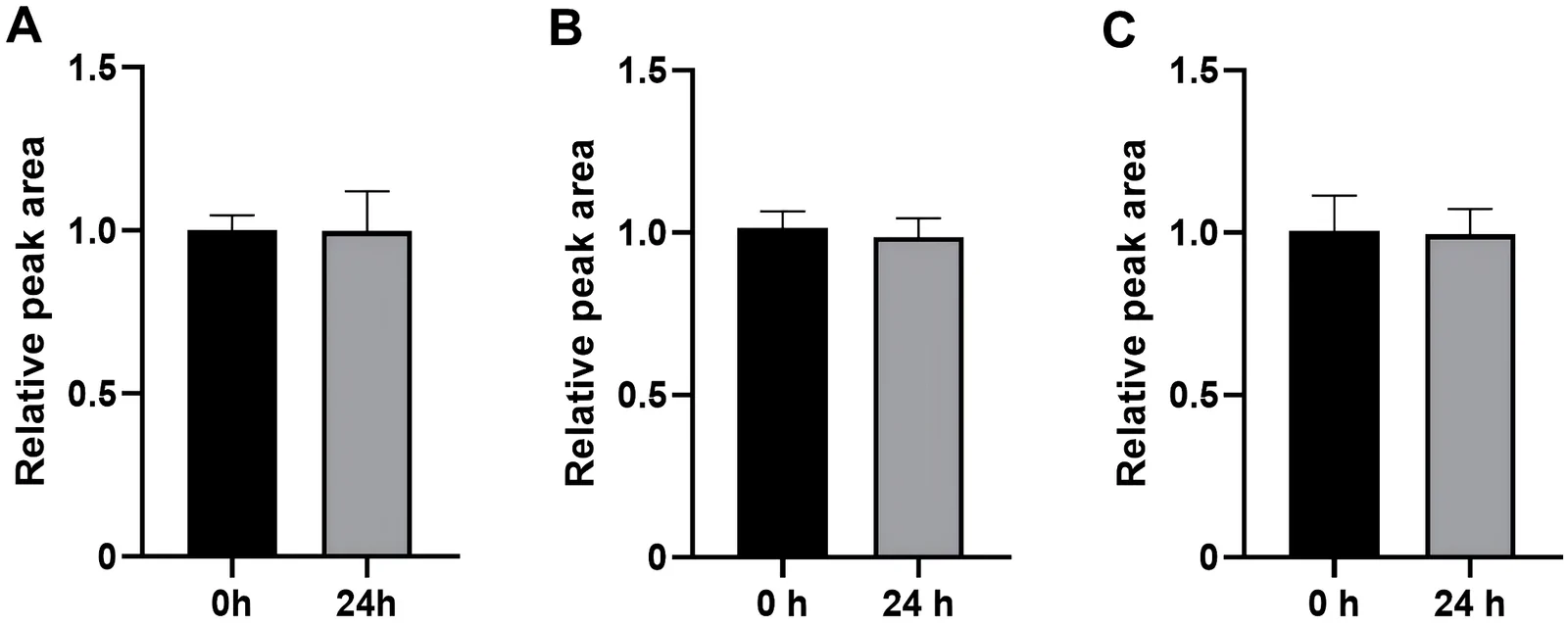
Relative quantitative of ticagrelor. Mean concentration of ticagrelor in mGAM medium (A), in the co-incubation system with human fecal suspensions (B), and rat fecal suspensions (C) at 0 h and 24 h. Data are represented as mean ± SD (n = 10).

### 3.4. Pharmacokinetics of ticagrelor and AR-C124910XX in pseudo-germ-free rats

To further evaluate whether the pharmacokinetics of ticagrelor were changed after the gut microbiota was eliminated *in vivo*, a pseudo-germ-free (PGF) rat model was constructed. Compared to the control group, the number of fecal microorganisms in PGF rats decreased by 86.61% in the PGF group, suggesting that the gut microbiota was extremely disrupted after the treatment of antibiotic cocktails (S3 Fig). The pharmacokinetic profiles of ticagrelor and its metabolite AR-C1249XX are shown in Fig 4, and the corresponding mean pharmacokinetic parameters are listed in Table 3 and Table 4. In oral study, no obvious changes were identified in the main pharmacokinetic parameters of ticagrelor and AR-C124910XX between control and PGF group, except for slight increases in MRT_(0–24)_, MRT_(0-∞)_ of ticagrelor and MRT_(0–24)_, MRT_(0-∞)_, and t_1/2_ of AR-C124910XX after microbiota depletion (Figs 4A and B, Table 3). Moreover, no significantly different pharmacokinetic parameters of ticagrelor were found after single intravenous administration (Fig 4C, Table 4), only minor increases in MRT_(0–24)_, MRT_(0-∞)_, and t_1/2_ of AR-C124910XX were identified in PGF rats (Fig 4C, Table 4). Combined with the above finding that the gut microbiota cannot directly metabolize ticagrelor, these results suggest that the gut flora has less influence on the pharmacokinetics of ticagrelor.

**Table 3:** Summary pharmacokinetic parameters of ticagrelor and AR-C124910XX after single intragastric administration in control and PGF rats.

Parameters	Ticagrelor		AR-C124910XX	
	Control	PGF	Control	PGF
AUC_(0–24)_ (ng/mL*h)	7862 ± 2325	8369 ± 3781	6253 ± 2086	5745 ± 1848
AUC_(0-∞)_ (ng/mL*h)	7936 ± 2354	8552 ± 3957	6294 ± 2093	5860 ± 1912
C_max_ (ng/mL)	937 ± 284.7	771.4 ± 293.0	542.3 ± 166.8	464.4 ± 118.8
T_max_ (h)	3.313 ± 1.831	3.625 ± 1.408	4.428 ± 1.134	5.857 ± 1.435
t_1/2_ (h)	3.451 ± 0.6456	3.981 ± 0.9065	3.228 ± 0.1872	3.849 ± 0.4740**
MRT_(0–24)_ (h)	5.411 ± 0.638	6.154 ± 0.577*	6.217 ± 0.2213	6.782 ± 0.4137**
MRT_(0-∞)_ (h)	5.612 ± 0.8203	6.588 ± 0.9008*	6.365 ± 0.2583	7.196 ± 0.6380**
CL (mL/h/kg)	2520 ± 1018	2555 ± 1266	3296 ± 1630	3347 ± 1027
Vd (mL/kg)	12.45 × 10^3^ ± 5001	14.13 × 10^3^ ± 6131	15.40 × 10^3^ ± 7545	18.30 × 10^3^ ± 4815

C_max_: maximum plasma concentration; T_max_: time to reach C_max_; AUC_(0–48)_ or AUC_(0-∞)_: area under the plasma concentration-time curve from zero to 48 h or infinity; t_1/2_: half-life of drug elimination; MRT: mean residence time; CL: clearance rate; Vd: apparent volume of distribution. Each value represents the mean ± SD. * *P* < 0.05, ** *P* < 0.01, *** *P* < 0.001 *vs.* the control group.

**Table 4:** Summary pharmacokinetic parameters of ticagrelor and AR-C124910XX after single intravenous administration in control and PGF rats.

Parameters	Ticagrelor		AR-C124910XX	
	Control	PGF	Control	PGF
AUC_(0–24)_ (ng/mL*h)	3144 ± 1120	3696 ± 623.1	924.0 ± 140.7	1031 ± 221.8
AUC_(0-∞)_ (ng/mL*h)	3210 ± 1155	3702 ± 634.6	926.9 ± 141.0	1037 ± 223.4
C_max_ (ng/mL)	1116 ± 218.7	1097 ± 160.3	113.4 ± 27.92	106.0 ± 38.06
T_max_ (h)	0.25 ± 0	0.25 ± 0	1.938 ± 1.652	2.438 ± 1.912
t_1/2_ (h)	2.363 ± 0.8722	2.713 ± 0.2586	2.761 ± 0.5652	3.331 ± 0.3498*
MRT_(0–24)_ (h)	2.728 ± 0.6905	3.186 ± 0.1821	4.410 ± 0.4023	4.785 ± 0.1977*
MRT_(0-∞)_ (h)	2.916 ± 0.5765	3.224 ± 0.1952	4.487 ± 0.4679	4.941 ± 0.2520*
CL (mL/h/kg)	1042 ± 350.0	830.0 ± 131.0	3301 ± 488.0	3032 ± 743.0
Vd (mL/kg)	3444 ± 1228	3236 ± 520.0	13.20 × 10^3^ ± 3753	14.57 × 10^3^ ± 3929

Cmax: maximum plasma concentration; Tmax: time to reach Cmax; AUC(0–48) or AUC(0-∞): area under the plasma concentration-time curve from zero to 48 h or infinity; t1/2: half-life of drug elimination; MRT: mean residence time; CL: clearance rate; Vd: apparent volume of distribution. Each value represents the mean ± SD. * P < 0.05, ** P < 0.01, *** P < 0.001 vs. the control group.

**Fig 4:**
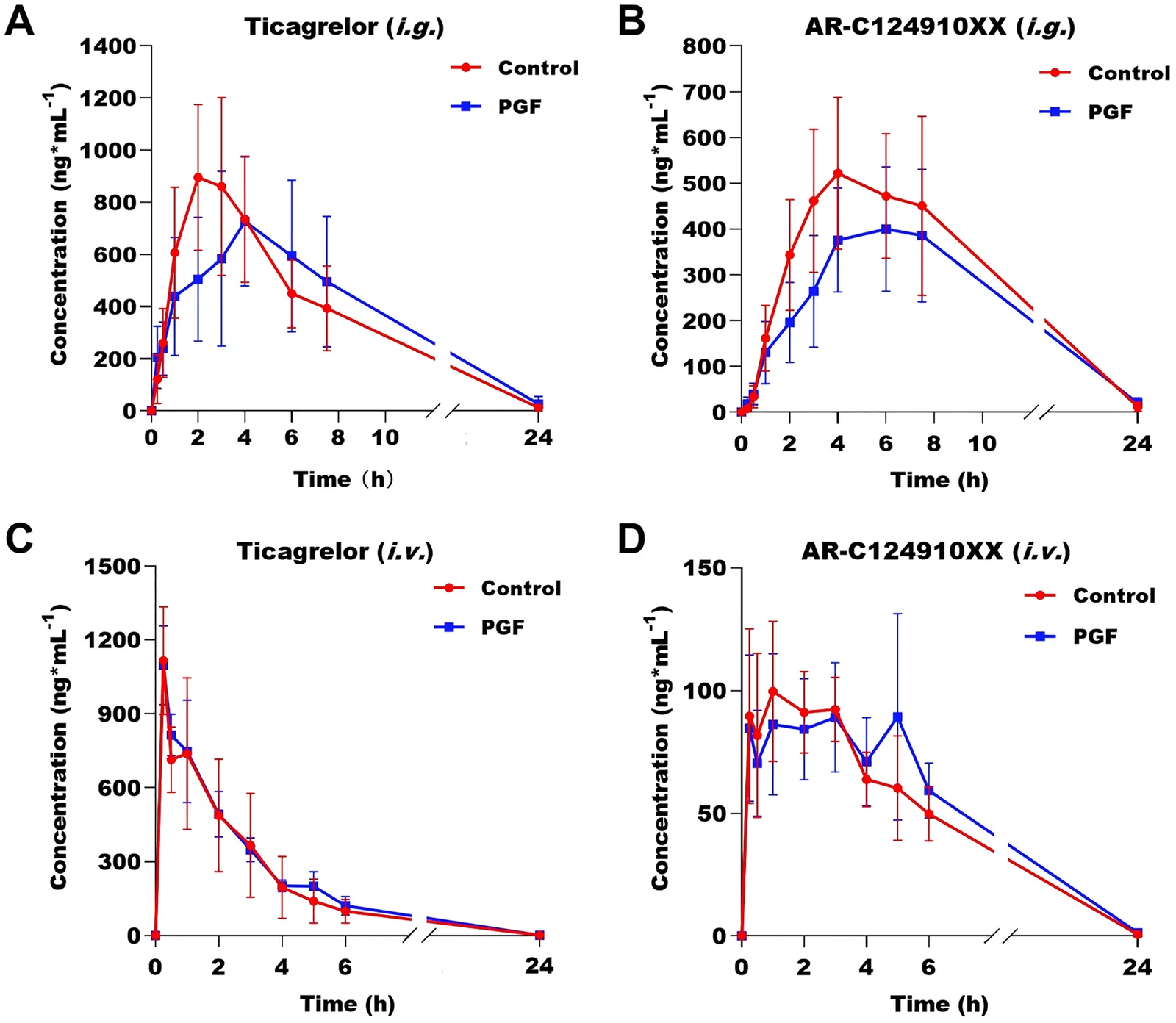
The plasma concentration-time curve of ticagrelor and AR-C124910XX in rat plasma after single intragastric (A&B) and intravenous (C&D) administration of ticagrelor. Data are represented as mean ± SD (n = 8).

## 4. Discussion

Compared with traditional antiplatelet drugs, ticagrelor is recommended as a first-line drug for the treatment of STEMI due to its rapid and stable onset of action. However, 20% of STEMI patients have no obvious response to ticagrelor, showing a delayed response. Individual differences in pharmacokinetic parameters have been proven to be one of the important factors affecting drug efficacy. In our present study, plasma concentrations of ticagrelor and its active metabolite AR-C124910XX were detected by recruiting healthy volunteers and found that the AUC_(0–24)_ and AUC_(0-∞)_ of ticagrelor and AR-C124910XX were 3-fold different between individuals, suggesting that there are large individual differences in the population pharmacokinetics of ticagrelor, which may affect drug efficacy.

Accumulating studies have focused on the influence of gut microbiota on drug pharmacokinetics and pharmacodynamics, which can alter drug bioavailability and efficacy either directly by producing active enzymes or indirectly by affecting host transporters or metabolizing enzymes through their metabolites [8]. However, most of the previous studies on the pharmacokinetics of ticagrelor only focused on the host gene polymorphisms but failed to well explain the individual pharmacokinetic differences, while ignoring the important role of the gut microbiota. In this study, we hypothesized that the pharmacokinetic differences in ticagrelor were likely due to the changes in the gut microbiota of different individuals.

By 16S rRNA sequencing, we analyzed the compositions of gut microbiota of individuals with different ticagrelor bioavailability. However, no significant correlation was found between gut microbiota and the pharmacokinetics of ticagrelor, suggesting that the gut microbiota may not be the main cause of individual differences in the pharmacokinetics of ticagrelor. At the same time, fecal samples were employed from humans and rats to primarily explore the bioconversion effects of intestinal bacteria on ticagrelor, and no direct metabolism was found. However, the existence of bioaccumulation of ticagrelor by bacteria remains to be further investigated.

To further study the effect of gut microbes on the pharmacokinetic parameters of ticagrelor *in vivo*, a microbiota-depleted PGF model was constructed using antibiotics, which has been widely used to investigate the role of gut microbiota in specific conditions [19], and subsequently evaluated the pharmacokinetic profiles of ticagrelor under oral and intravenous administration. As the results showed, apparently increased MRT_(0–24)_ and MRT_(0-∞)_ of ticagrelor were observed in PGF rats under oral administration compared to intravenous administration, indicating that the effects of microbiota-depletion on ticagrelor were mainly in the intestine. This may be related to decreased expression of the intestinal efflux transporter P-glycoprotein (P-gp), which leads to reduced transport of ticagrelor from the blood to the intestine, thereby prolonging the retention time of ticagrelor in the body [20]. A Previous study reported that the expression of Cyp3a11 (CYP3A4 homologous enzyme) was reduced by 60% and 86% in the duodenum and ileum of mice after microbiota depletion [21], resulting in decreased metabolism of ticagrelor in the intestine and increase blood absorption.

The association between ticagrelor and gut microbiota has been reported before, which enrolled 65 patients with high platelet reactivity (HPR) and 90 controls with normal platelet reactivity (NPR). Pharmacokinetic evaluation showed that plasma concentrations of ticagrelor and its metabolite AR-C124910XX were lower in HPR than in controls, while HPR patients had higher microbial abundance and diversity. This result is similar to our findings that the bioavailability of ticagrelor was relatively low in control rats (higher microbial abundance and diversity) [16]. However, the effects of antibiotics on drug-processing genes are complex and may induce other unrecognized drug transporter proteins or drug-metabolizing enzymes [22], resulting in inapparent changes in plasma ticagrelor concentrations. Therefore, our findings favor that antibiotics have no distinct effect on the pharmacokinetics of ticagrelor.

There are some limitations in the current study. Firstly, insufficient sample size may be an important factor affecting the effectiveness of explaining individual differences. Secondly, the inclusion of healthy subjects may not fully reflect the patients who will be using ticagrelor. Thirdly, drug pharmacokinetic alterations are only clinically relevant if platelet reactivity is also altered, but the platelet reactivity of ticagrelor in the presence or absence of gut microbiota was not evaluated in our study. Fourthly, we acknowledge that the single‑dose pharmacokinetic results cannot fully replicate steady‑state exposure; our findings serve as a basis for further multiple‑dose PK/PD investigations rather than a direct prediction of chronic dosing effects. Thus, further investigations are needed to disclose the effects of the gut microbiota in the clinical treatment of ticagrelor in larger sample sizes and different populations using ticagrelor, with extended dosing regimens and inclusion of pharmacodynamic assessments.

## 5. Conclusion

Overall, our investigation based on the pharmacokinetic assessment of ticagrelor in healthy volunteers and microbiota-depleted PGF rats suggested that gut microflora may not be the primary cause of individual differences in the pharmacokinetics of ticagrelor. Ticagrelor can’t be directly metabolized by intestinal bacteria *in vitro*. However, as the present study did not evaluate other potential mechanisms of drug–drug interactions between ticagrelor and antibiotics (e.g., effects on drug‑metabolizing enzymes or transporters), our findings do not completely exclude the possibility of indirect interactions. Therefore, we suggest that no dosage adjustment is specifically required based on microbiota‑mediated pathways; nevertheless, prudent safety monitoring is still warranted when ticagrelor is co‑administered with antibiotics.

## Supporting information

S1 Table Clinical characteristics of in healthy subjects enrolled in the study. (DOCX)

S1 Fig Cluster analysis of subjects based on pharmacokinetic parameters of ticagrelor. Group H included subjects with higher AUC_(0–48)_ and AUC_(0-∞)_. Group H included subjects with lower AUC_(0–48)_ and AUC_(0-∞)_. Group H: n = 12. Group L: n = 10.
(TIF)

S2 Fig The plasma concentration-time curve of ticagrelor and AR-C124910XX in healthy subjects. Data are represented as mean ± SD. Group H: n = 12. Group L: n = 10.
(TIF)

S3 Fig Fecal DNA concentration of the normal rats (control) and pseudo germ free rats (PGF). Data are represented as mean ± SD. **P < 0.01 vs. control group (n = 8).
(TIF)
