# Supplementary material for: Ticagrelor and gut microbiota: Absence of pharmacokinetic interaction in vitro and in vivo studies
Source: PLoS One. 2026 Oct 1;21(10):e0359660. doi: 10.1371/journal.pone.0359660 (PMC13630236; doi:10.1371/journal.pone.0359660)
Supplement: Supplementary file 1 [file pone.0359660.s001.docx]

**Table S1.** Clinical characteristics of in healthy subjects enrolled in the study.

| **Characters** | **All participants**  **(n, %)** | **Group H**  **(n, %)** | **Group L**  **(n, %)** |
| --- | --- | --- | --- |
| Gender (Female） | 2 (10%) | 2 (20%) | 0 (0%) |
| Age (years) | 27.4 ± 5.3 | 27.9 ± 5.9 | 26.7 ± 4.7 |
| **Anthropometric marker** |  |  |  |
| Weight (kg) | 61.5 ± 7.6 | 61.3 ± 7.5 | 61.8 ± 8.0 |
| Height (cm) | 167.2 ± 7.8 | 163.8 ± 6.7 | 171.2 ± 7.3 |
| BMI | 22.0 ± 2.4 | 21.7 ± 2.1 | 21.1 ± 2.5 |
| **Laboratory test** |  |  |  |
| PLT (109/L) | 221.3 ± 39.1 | 223.0 ± 35.9 | 219.2 ± 44.6 |
| MPV (fL) | 8.56 ± 0.71 | 8.63 ± 0.49 | 8.48 ± 0.94 |
| PCT (%) | 0.19 ± 0.02 | 0.19 ± 0.02 | 0.18 ± 0.03 |
| PDW (%) | 16.50 ± 0.48 | 16.62 ± 0.46 | 16.36 ± 0.48 |

Each value represents the mean ± SD.
